# A Comparison of the Prevalence of Gastrointestinal Parasites in Wild Boar (*Sus scrofa* L.) Foraging in Urban and Suburban Areas

**DOI:** 10.3390/ani14030408

**Published:** 2024-01-26

**Authors:** Bogumiła Pilarczyk, Agnieszka Tomza-Marciniak, Renata Pilarczyk, Lidia Felska-Błaszczyk, Małgorzata Bąkowska, Jan Udała, Marta Juszczak-Czasnojć

**Affiliations:** 1 Department of Animal Reproduction Biotechnology and Environmental Hygiene, Faculty of Biotechnology and Animal Husbandry, West Pomeranian University of Technology, 71-270 Szczecin, Poland; bogumila.pilarczyk@zut.edu.pl (B.P.); malgorzata.bakowska@zut.edu.pl (M.B.); jan.udala@zut.edu.pl (J.U.); marta.juszczak-czasnojc@zut.edu.pl (M.J.-C.); 2 Laboratory of Biostatistics, Faculty of Biotechnology and Animal Husbandry, West Pomeranian University of Technology, 71-270 Szczecin, Poland; renata.pilarczyk@zut.edu.pl; 3 Department of Animal Anatomy, Faculty of Biotechnology and Animal Husbandry, West Pomeranian University of Technology, 71-270 Szczecin, Poland; lidia.felska-blaszczyk@zut.edu.pl

**Keywords:** wild boar, parasites, *Eimeria* sp., *Ascaris suum*, *Oesophagostomum dentatum*

## Abstract

**Simple Summary:**

Parasitological examinations of wild boar foraging in urban and suburban areas revealed the presence of a mixed infection of coccidia and gastrointestinal nematodes. The parasites *Eimeria debliecki*, *E. suis*, *E. polita*, *E. scabra* and *Isospora suis* and two species of nematodes, *Ascaris suum* and *Oesophagostomum dentatum*, were observed in both analysed groups of animals. Wild boar from the city were characterised as having a higher prevalence of total *Eimeria* and a lower prevalence of noted species of nematodes compared to those from the suburban area. Since the wild boars were mainly infected with *Eimeria*, it should be assumed that they may pose a real health threat to farm pigs and other farm animals for which *Eimeria* is a pathogenic parasite. The occurrence of coccidiosis leads to serious health problems and economic losses for breeders. Although the prevalence of *A. suum* was low, it should be taken into account that this nematode is able to both infect and complete their life cycle in humans.

**Abstract:**

The aim of this study was to compare the species composition of gastrointestinal parasites in wild boar feeding in the city of Szczecin with those in its suburban area, as well as to determine the prevalence and intensity of this parasite infection. The intestines and stomachs of 57 wild boars were supplied by a municipal hunter from the city of Szczecin. Both analysed groups of animals were infected with the following parasites: *Eimeria debliecki*, *E. suis*, *E. polita*, *E. scabra, Isospora suis*, *Ascaris suum* and *Oesophagostomum dentatum*. Wild boar from the city were characterised as having a significantly higher prevalence of total *Eimeria* (*p* = 0.04) and a lower prevalence of noted species of nematodes (*p* = 0.15) compared to those from the suburban area. Since the wild boars were mainly infected with *Eimeria*, it should be assumed that they may pose a real health threat to farm pigs and other farm animals for which *Eimeria* is a pathogenic parasite. The occurrence of coccidiosis leads to serious health problems and economic losses for breeders. Although the prevalence of *A. suum* was low, it should be taken into account that this nematode is able to both infect and complete their life cycle in humans.

## 1. Introduction

Although the principal habitat of the wild boar (*Sus scrofa*) is the forest, cities with large green areas, such as urban forests, city parks, and city squares, can also provide attractive places for them to rest and forage. Urban areas also lack large predators and hunters; offer easier access to food from city inhabitants, unsecured rubbish containers, and unfenced plots of land; and provide more favourable conditions for surviving winter, which is milder in the city. As such, wild boar are increasingly attempting to settle within urban agglomerations and are quickly adapting to new environmental conditions and to the presence of humans. While foraging in an urban area, these animals are able to overcome their fear of humans and move freely in between buildings. It is believed that wild boar are now present in more than 80 cities in Poland [1], indicating that the benefits of living close to humans outweigh the risks associated with urbanisation.

The wild boar population in Poland decreased significantly between 2014 and 2022 due to ASF, falling from 285,000 individuals in the 2013/2014 hunting season to only 67,900 in the 2020/2021 hunting season. Reducing the feral pig population in forest and urban and suburban environments also reduces the number of animals infected with parasites, and hence the risk of parasite transmission between wild boars. A higher population density, i.e., a greater abundance of wild boar, is associated with a faster spread of parasitic disease among animals. Since 2017, the Ministry of the Environment has abolished the protection period for wild boar, and wild boar can be hunted all year round, even sows leading dependent piglets. The resulting decline in wild boar populations, together with their settlement in urban areas, may affect the prevalence and intensity of parasite infection among them.

However, no studies have yet compared the prevalence of parasites between wild boars living in urban areas and suburban areas in Poland. As such, our findings are the first to present the species composition of the parasitofauna of wild boar foraging in urban and suburban areas in Szczecin, Poland and determine the prevalence and intensity of infection.

The high prevalence of infection and high parasite diversity, combined with the high reproduction rate and migration of wild boar, allow the environment to remain highly contaminated with dispersion forms. The profile of specific parasites present in wild boar populations tends to reflect the environmental conditions in the local area. Urban areas differ from suburban areas in terms of microclimate—mainly temperature and humidity; these factors significantly affect the survival and life cycle of the environmental forms of parasites and their spread in the environment. For example, an increase in temperature can change the ranges of certain parasite species, affecting their ability to survive in new regions. Changing the structure of the ecosystem can create new conditions for growth or reduce the availability of some parasites.

As urban wild boar tend to consume a lower proportion of food components involved in the parasite development cycle, we hypothesise that wild boar (*Sus scrofa*) populations foraging in urban areas have a lower diversity, prevalence, and intensity of gastrointestinal parasite infection compared to suburban wild boar populations.

The aim of this study was to compare the species composition of gastrointestinal parasites in wild boars feeding in the city of Szczecin with those in its suburban area, as well as to determine the prevalence and intensity of infection of these parasites.

## 2. Materials and Methods

### 2.1. The Natural Characteristics of Szczecin

Due to the fact that Szczecin covers an extensive area, around 30,000 ha, with a relatively high proportion of green areas (41.6%), the area is a very attractive habitat for wild boar. Its boundaries encompass 16 urban parks and urban forests with an area of 2800 ha; these include Arkona Forest Park, Głębokie Forest Park, and Zdroje Forest Park. In addition, there are more than 80 Allotment Gardens within and around the city, occupying 2500 ha. These serve as nurseries and attractive “feeding grounds” for wild boar. In addition, the city is surrounded by important habitats for wildlife, such as the three primeval forests Wkrzańska, Goleniowska, and Bukowa, as well as the Lower Oder Valley. The suburban area where wild boars came from is located east of Szczecin, on the edge of the Wkrzańska Forest. Wild boar from the urban area came from the western part of Szczecin.

### 2.2. Materials

Research material in the form of wild boar gastrointestinal tracts was obtained from boars that were not specifically killed for these studies. The culling of wild boars was carried out as part of urban hunting activities related to controlling the wild boar population within the Szczecin metropolitan area and suburban area. This culling was performed under a signed agreement with the Szczecin City Office by the company, authorising and possessing all necessary permits for the culling of wild boars and the transportation of biological material. This agreement includes a provision specifying the method of wild boar acquisition, which is conducted using traps and then shooting at the capture site. The culling is conducted using semi-jacketed bullets, calibres 232 and 223, which do not exit the carcass. This culling is permitted by the regulations of the Animal Protection Act [2] and the Hunting Law [3].

**Figure 1:**
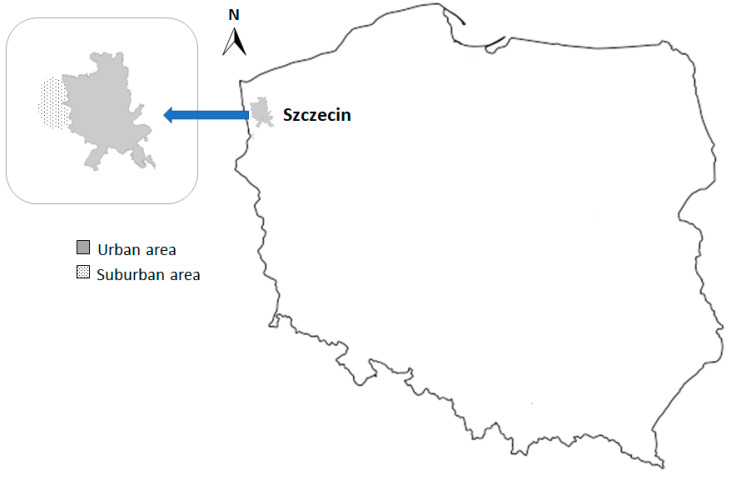
Location of the study site.

**Figure 2:**
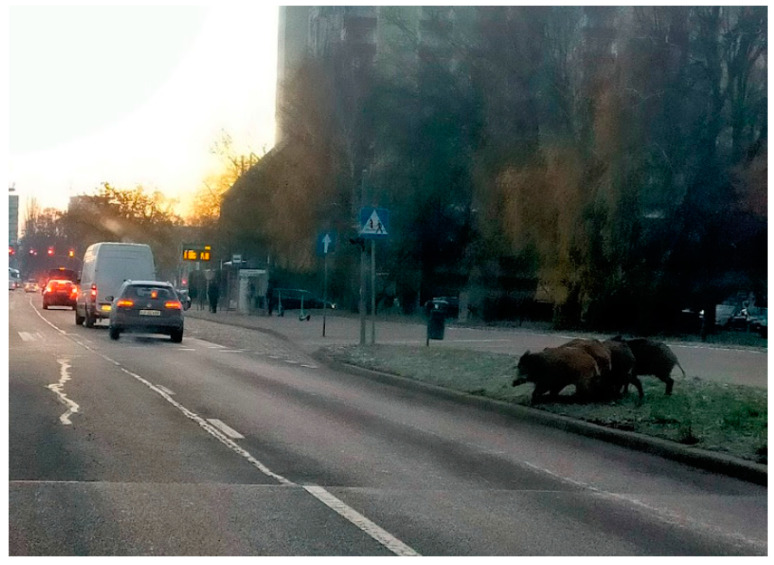
Wild boars in the city of Szczecin, on the Przyjaciół Żołnierza Street. This place is located in the western part of Szczecin, 2.9 km from the city centre.

The bodies of 56 wild boars (40 individuals from the urban area and 16 individuals from the suburban area) were supplied by a municipal hunter from the city of Szczecin (Figure 1 and Figure 2). The intestines and stomach were removed and transported to the Department of Animal Reproduction Biotechnology and Environmental Hygiene, where they were tested for internal parasites. Sex and body weight (kg) were determined.

### 2.3. Research Methods

Parasitological dissection was carried out according to Dróżdż [4]. The intestines were examined by sedimentation and counting (SCT) [5]. Each bowel was opened along its entire length and examined macroscopically for large worms. The digestive tract was then divided into three sections (stomach, small intestine, and large intestine), which were then cut lengthwise, and the entire contents were washed away. The blunt side of the scalpel was used to remove any remaining contents, and the intestinal surfaces were examined. Three mucosal swabs were prepared from each individual section and placed in Petri dishes; a thin layer of the smear was examined under a microscope at 120× magnification.

The gastrointestinal contents were moved to 4 L beakers and mixed with water. The resulting mixture was collected in 400 mL beakers and tested using the decanting method. Samples were taken from the mixture, divided into small portions, and examined under a microscope [6]. Adult nematodes were identified by morphological examination (shape, dimensions, structural features) according to Thienpont et al. [7], Foreyt [8], and Anderson [9]. The species composition of *Eimeria* protozoa was determined using the Pellerdi key [10] on the basis of oocyst morphology, viz. their size, shape, colour, presence or absence of micropyles, presence or absence of residual bodies, and sporulation time.

The prevalence of infection was determined via postmortem examination for gastrointestinal nematodes (adult individuals) and via coproscopic examination for the coccidia. In addition, faeces were collected from the terminal ileum to determine the species composition of *Eimeria* protozoa and the intensity of the coccidial infection. Coproscopic surveys were carried out using (1) the Willis–Schlaaf flotation method, comprising sodium chloride flotation (d = 1.200 g/mL), and (2) the McMaster quantitative method with counting chambers (correction factor = 100) [7].

This study was complemented by an oocyst culture in a humidity chamber at 24 to 26 °C. A 2.5% aqueous solution of potassium dichromate (K_2_Cr_2_O_7_) was added to prevent mould growth.

The prevalence and mean intensity of infection with particular parasite species were calculated using the formula described by Margolis et al. [11]. In the case of coccidia, the intensity of infection was expressed as OPG—number of oocysts in one gram of faeces—and in the case of nematodes—as a number of adult parasites in one host.

### 2.4. Statistical Analysis

The results were analysed using Statistica 13.3 (TIBCO Software Inc., Palo Alto, CA, USA). The intensity of the infection of individual parasite species between host groups was compared using the χ^2^ test, while the intensity of infection was compared using the non-parametric Mann–Whitney test.

## 3. Results

The coproscopic examinations revealed the presence of a mixed infection of coccidia and gastrointestinal nematodes. Five species of protozoan were found in animals from both areas, viz. *E. debliecki*, *E. suis*, *E. polita*, *E. scabra*, and *Isospora suis*, and two species of nematodes, *Ascaris suum* and *Oesophagostomum dentatum* (Table 1). The wild boar from the urban area were characterised by a significantly (*p* < 0.05) higher prevalence of *E. suis* infection (25% vs. 0%, *p* = 0.03) and a total prevalence of total *Eimeria* (47.5% vs. 18.8%, *p* = 0.04) compared to those from the suburban area. In the case of nematodes, a higher prevalence was recorded in wild boars from the suburban area than from the urban area, but the differences were not statistically significant (Table 1).

As few infected individuals were found in the area outside the city, it was not possible to identify differences in the intensity of protozoan or nematode infection (Table 2).

Host age only appeared to have a significant influence on the prevalence of combined *Eimeria* (χ^2^ = 4.0; *p* = 0.04) and the nematode *Ascaris suum.* In both cases, significantly higher prevalence was noted in individuals with higher body weights (40–70 kg) (*p* < 0.05).

Host sex only appeared to have an influence on the prevalence of *E. scabra* (χ^2^ = 4.7, *p* = 0.03). No significant differences in the mean intensity of protozoan (Z = 0.07, *p* = 0.94) and nematode (Z = 7.00, *p* = 1.00) infection were noted between male and female hosts (Table 3).

Host body weight only seemed to have a significant influence on the prevalence of combined protozoan infection (χ^2^ = 4.2, *p* = 0.04) and for *Ascaris suum (*χ^2^ = 8.4, *p* = 0.004). In both cases, individuals with higher body weights (40–70 kg) were characterised by a significantly (*p* < 0.05) higher prevalence (Table 3). Significant differences in the intensity of *E. debliecki* and *E. suis* infection and combined protozoan species were found between the weight groups, with a higher intensity noted in lighter animals, i.e., up to 40 kg (U = 0.0, *p* < 0.01) (Table 4).

## 4. Discussion

Parasites can adversely affect the health of wild boar, causing a variety of illnesses, debilitation, or even death [12]. However, regular testing allows the health status of the feral pig population to be monitored and for appropriate preventive measures to be taken. The survival of young piglets and weaners is largely linked to the prevalence of parasites, especially *Eimerian* protozoa. At high levels of infection, these younger pigs become thin, lethargic, and less mobile. They also lose their appetite and develop diarrhoea, with blood possibly appearing in faeces [13,14]. Diarrhoea results in a decrease in weight gain and even death.

Relatively few papers have examined the presence of *Eimeria* protozoa in wild boars. This is probably due to the fact that it is difficult to monitor infections in free-living animals. In this study, oocysts of *Eimeria* were found in the wild boars studied. It is important to monitor the infection rate of *Eimeria* sp. in wild boar to maintain healthy populations, especially among young individuals. Our findings indicate that coccidia were the most common parasites in the wild boar studied (39.3%). The prevalence of *Eimeria* infections in wild boars from the urban area was 47.5%, and it was more than double that in the suburban area (18.8%). This high prevalence in the city may be due to the excessive density of hosts and the fact that wild boars return to places where they find food. Another study conducted in Northwestern Poland recorded an infection rate of 58.8% in forest-dwelling wild boar [15]. Other authors have also identified *Eimeria* protozoa in these animals, with extensities of 33.33% in Italy [16], 7.5% in Bulgaria [17], and 3% in Russia [18]. Such variation can be related to environmental factors (e.g., warmer winters). The prevalence of infection was found to be as high as 92.5% among wild boar kept in reserves covering areas of up to 40 km^2^ [19].

Oocysts are highly resistant to external environmental conditions, making it possible for them to survive and accumulate in the environment for many months while retaining the ability to infect [20].

Our findings indicate that individuals with a higher body weight (40–70 kg) tended to have a higher intensity of infection. Wild boar with a larger body mass may need a larger area to forage. This allows them to move greater distances into new areas in search of food, thus increasing the risk of contact with other animals that may carry parasites. Heavier wild boar may also be older, which entails a longer exposure to parasites and potentially more contact with other animals and areas, further increasing the chance of infection.

In our study, significant differences were found between the wild boar weight groups with regard to the intensity of infection of *E. debliecki* and *E. suis*, as well as the total intensity of infection of all protozoans. A significantly higher mean intensity of infection was noted among lighter wild boar, i.e., those weighing up to 40 kg. These lighter animals may be younger and also more susceptible to *Eimeria* infection, as their immune system may be less developed. Such immunity, acquired with age, plays a key role in host resistance, which develops through continuous exposure to varying degrees of coccidial invasion. However, while this acquired resistance does not completely eliminate infection, it does allow for effective reduction in coccidia proliferation in the digestive tract [21].

*Ascaris suum* nematodes are intestinal parasites of wild boar and pigs and are known to be transmitted through soil. Its prevalence, and of *Ascaris* in general, is enhanced by its high reproductive potential (200,000 eggs/day), with its eggs being highly resistant to harmful external agents, exhibiting their longevity [22]. Its occurrence in wild boar has been found to vary according to the location, population, and age of the host [12]. Evidence suggests that *A. suum* may, in fact, be the same species as *A. lumbricoides*, although the pair are reproductively isolated [23,24,25]. The species is of zoonotic significance, as studies have found interspecies transmission to occur between pigs and humans living in close proximity or where pig manure is used as fertiliser for vegetables intended for human consumption [26,27].

In this study, it was found that the prevalence and intensity of *A. suum* was relatively low, being present in only 5% of wild boars in the urban area and 12.5% of wild boars in the suburban area. This low prevalence of infection may be due to the fact that the city environment inhabited by the wild boars is not a typical habitat for parasites such as *A. suum*, which can limit direct exposure. The wild boar in this study live in small groups, constantly migrating from place to place around the city looking for food, which can reduce exposure to parasite infection. Urban areas are, to varying degrees, rich in food or organic waste, which may be less contaminated with parasite eggs. Obtaining food in urban areas does not require foraging as frequently as in suburban areas, which reduces contact with soil that may be contaminated with *A. suum*. Moreover, urban areas are characterized by higher temperatures and lower air humidity, and these are parameters that favour egg inactivation [28].

The prevalence of *A. suum* infection in suburban wild boars (7.1%) is lower than previously noted among suburban wild boars in Belgrade (9.37%) [29]. *A. suum* has been found in wild boar in Poland [30], Spain [31], Brazil [24], Russia [18], and Finland [32]. Also, our data indicate that, while *A. suum* was not present in lighter animals, i.e., under 40 kg, it was found in 21.1% of individuals in the 40–70 kg range. As such, the latter were a potential source of infection for smaller animals and for the contamination of the area via eggs.

Regarding nematode infection, a much lower prevalence in wild boars from the suburban area (25%) was noted compared to suburban wild boars of Belgrade (68.08%) [29]. In this study, in Szczecin, the prevalence of infection was more than 2.5 times lower (25%) in the suburban wild boars and almost seven times lower (10%) in wild boars from the urban area. However, neither study found any significant differences in the prevalence of nematode infection between males and females. The lack of differences between sexes may be explained by the fact that gastrointestinal parasites in wild boars are spread by consuming contaminated food or water, direct contact with an infected individual, and contact with contaminated objects. And none of these pathways are related to the sex of the animal.

The wild boars from the urban area were less likely to be infected with *Oesophagostomum dentatum* (prevalence 5%) than those from the suburban area (18.8%). This variation may be due to the low survival rate of *O. dentatum* larvae in the soil in conditions of high air temperature and low humidity. Urban areas tend to absorb and retain heat more than suburban areas due to the presence of materials such as concrete and asphalt. The temperature can be raised further by human activities, such as traffic and the use of air conditioning, and there is often little greenery to help regulate temperature [33]. As a result, these *heat islands* can be a few degrees hotter than surrounding areas. This urban heat island effect may well have been responsible for the low prevalence in Szczecin; Roepstorff et al. [34] found *O. dentatum* larvae to have a low survival rate in the soil of pastures used by pigs during the hottest and driest summers and coldest winters.

## 5. Conclusions

Research on the gastrointestinal parasites of wild boars in urban and suburban areas is crucial, as it helps to better understand the dynamics of parasites in these types of environments. In this study, we found that the wild boar from the urban area were characterised by a significantly higher prevalence of total *Eimeria* (*p* = 0.04) and a lower prevalence of observed species of nematodes (*p* = 0.15) compared to those from the suburban area.

The results obtained from these studies indicate that wild boars should be considered potential reservoirs of parasites—especially for other animals but also for humans. Because Eimeria sp. was the main parasite found in wild boar, it should be assumed that these animals may pose a real health threat to farm pigs and other farm animals for which *Eimeria* is a pathogenic parasite. Although the prevalence of *A. suum* was low, it should be taken into account that this nematode is able to infect and complete their life cycle in humans. Therefore, the presence of wild boar in urban areas may lead, over the years, to a systematic increase in the density of nematode eggs in the environment and an increase in the risk of infection in humans.

Understanding the species composition of parasites present in wild boars from urban areas can be useful in planning strategies for managing the wild boar population and also for taking actions aimed at protecting the health of residents in these areas.

Considering the fact that wild boars can carry parasites harmful to the health of humans and animals, it seems necessary to implement a periodic monitoring program for internal parasite infections in wild boars within city limits.

## Figures and Tables

**Table 1:** The prevalence of gastrointestinal parasite infection in the wild boars studied, in relation to sex, body weight, and region.

	*E. debliecki*		*E. suis*		*E. polita*		*E. scabra*		*Isospora suis*		*Eimeria* Total		*O.* *dentatum*		*Ascaris suum*		Nematodes Total	
	n	%	n	%	n	%	n	%	n	%	n	%	n	%	n	%	n	%
Sex																		
female (N = 33)	63	18.2	6	18.2	1	3.0	6	18.2	1	3.0	12	36.4	2	6.1	3	9.1	53	15.2
male (N = 23)	4	17.4	4	17.4	4	17.4	0	0.0	2	8.7	10	43.5	3	13.0	1	4.4	3	13.0
	χ^2^ = 0.01 *p* = 0.94		χ^2^ = 0.01 *p* = 0.94		χ^2^ = 3.4 *p* = 0.06		χ^2^ = 4.7 *p* = 0.03		χ^2^ = 0.9 *p* = 0.36		χ^2^ = 0.3 *p* = 0.59		χ^2^ = 0.8 *p* = 0.37		χ^2^ = 0.5 *p* = 0.50		χ^2^ = 0.05 *p* = 0.82	
Body mass																		
< 40 kg (N = 37)	6	16.2	5	13.5	3	8.1	3	8.1	3	8.1	11	29.7	3	8.1	0	0.0	3	8.1
40–70 kg (N = 19)	4	21.1	5	26.3	2	10.5	3	15.8	0	0.0	11	57.9	2	10.5	4	21.1	5	26.3
	χ^2^ = 0.2 *p* = 0.65		χ^2^ = 1.4 *p* = 0.24		χ^2^ = 0.09 *p* = 0.76		χ^2^ = 0.8 *p* = 0.38		χ^2^ = 1.6 *p* = 0.20		χ^2^ = 4.2 *p* = 0.04		χ^2^ = 0.09 *p* = 0.76		χ^2^ = 8.4 *p* = 0.004		χ^2^ = 3.4 *p* = 0.06	
Region																		
urban area (N = 40)	9	22.5	10	25.0	4	10.0	4	10.0	3	7.5	19	47.5	2	5.0	2	5.0	4	10.0
suburban area (N = 16)	1	6.3	0	0.0	1	6.3	2	12.5	0	0.0	3	18.8	3	18.8	2	12.5	4	25.0
	χ^2^ = 2.1 *p* = 0.15		χ^2^ = 4.9 *p* = 0.03		χ^2^ = 0.2 *p* = 0.66		χ^2^ = 0.07 *p* = 0.78		χ^2^ = 1.3 *p* = 0.26		χ^2^ = 4.0 *p* = 0.04		χ^2^ = 2.7 *p* = 0.10		χ^2^ = 0.1 *p* = 0.32		χ^2^ = 2.1 *p* = 0.15	

total (N = 56)	10	17.9	10	17.9	5	8.9	6	10.7	3	5.4	22	39.3	5	8.9	4	7.1	8	14.3

n—number of infected animals; N—total number of animals included in the study.

**Table 2:** The prevalence and intensity of gastrointestinal parasite infection in wild boars according to region.

Parasite	Locality	n/N	Prevalence (%) (95% CI)	χ^2^ Test Value	Intensity of Infection *					
					Mean	GM	Median	Range	Mann–Whitney U-Test Value	
Eimeria										
*E. debliecki*	urban area	9/40	22.5 (12.1–37.7)	χ^2^ = 2.1 *p* = 0.15	367	267	300	100–900	-	
	suburban area	1/16	6.3 (0.01–30.3)		400	400	400	400–400		
*E. suis*	urban area	10/40	25.0 (14.0–40.4)	χ^2^ = 4.9 *p* = 0.03	425	331	425	100–800	-	
	suburban area	0/16	0.0 (0.0–22.7)							
*E. polita*	urban area	4/40	10.0 (3.4–23.6)	χ^2^ = 0.2 *p* = 0.66	125	93	75	50–300	-	
	suburban area	1/16	6.3 (0.01–30.3)		650	650	650	650–650		
*E. scabra*	urban area	4/40	10.0 (3.4–23.6)	χ^2^ = 0.07 *p* = 0.78	313	168	250	50–700	U = 3.0 *p* = 0.80	
	suburban area	2/16	12.5 (2.2–37.3)		350	245	350	100–600		
*Isospora suis*	urban area	3/40	7.5 (1.9–20.6)	χ^2^ = 1.3 *p* = 0.26	217	208	200	150–300	-	
	suburban area	0/16	0.0 (0.0–22.7)							
*Eimeria* total	urban area	19/40	47.5 (32.9–62.5)	χ^2^ = 4.0 *p* = 0.04	524	332	250	50–1800	Z = 0.24 *p* = 0.79	
	suburban area	3/16	18.8 (5.8–43.8)		583	402	650	100–1000		
Nematodes										
*Oesophagostomum dentatum*	urban area	2/40	5.0 (0.5–17.4)	χ^2^ = 2.7 *p* = 0.10	4	3	4	3–4	U = 2.5 *p* = 0.80	
	suburban area	3/16	18.8 (5.8–43.8)		3	2	3	1–5		
*Ascaris suum*	urban area	2/40	5.0 (0.5–17.4)	χ^2^ = 0.1 *p* = 0.32	2	2	2	1–3	U = 1.5 *p* = 0.67	
	suburban area	2/16	12.5 (2.2–37.3)		3	2	3	2–3		
Nematodes total	urban area	4/40	10.0 (3.4–23.6)	χ^2^ = 2.1 *p* = 0.15	3	2	3	1–4	U = 7.5 *p* = 0.89	
	suburban area	4/16	25.0 (9.7–50.0)		4	3	3	1–7		

n—number of infected animals; N—total number of animals included in the study. * OPG—oocyst per gram (protozoa) or number of adult parasites in the host (nematodes). GM—geometric mean.

**Table 3:** The prevalence and intensity of gastrointestinal parasite infection in wild boars according to the sex of the host.

Parasite	Sex	n/N	Prevalence (%) (95% CI)	χ^2^ Test Value	Intensity of Infection *				
					Mean	GM	Median	Range	Mann–Whitney U-Test Value
Eimeria									
*E. debliecki*	female	6/33	18.2 (8.2–34.8)	χ^2^ = 0.01 *p* = 0.94	333	245	250	100–900	U = 9.0 *p* = 0.61
	male	4/23	17.4 (6.4–37.7)		425	336	400	100–800	
*E. suis*	female	6/33	18.2 (8.2–34.8)	χ^2^ = 0.01 *p* = 0.94	358	285	225	150–800	U = 9.0 *p* = 0.61
	male	4/23	17.4 (6.4–37.7)		525	412	600	100–800	
*E. polita*	female	1/33	3.0 (0.01–16.7)	χ^2^ = 3.4 *p* = 0.06	50	50	50	50–50	-
	male	4/23	17.4 (6.4–37.7)		275	177	200	50–650	
*E. scabra*	female	6/33	18.2 (8.2–34.8)	χ^2^ = 4.7 *p* = 0.03	325	190	275	50–700	-
	male	0/23	0.0 (0.0–16.9)						
*Isospora suis*	female	1/33	3.0 (0.01–16.7)	χ^2^ = 0.9 *p* = 0.36	300	300	300	300–300	-
	male	2/23	8.7 (1.3–28.0)		175	173	175	150–200	
*Eimeria* total	female	12/33	36.4 (22.1–53.4)	χ^2^ = 0.3 *p* = 0.59	538	322	225	100–1800	Z = 0.07 *p* = 0.94
	male	10/23	43.5 (25.6–63.2)		525	366	625	50–900	
Nematodes									
*Oesophagostomum dentatum*	female	2/33	6.1 (0.7–20.1)	χ^2^ = 0.8 *p* = 0.37	4	3	4	3–4	U = 2.5 *p* = 0.80
	male	3/23	13.0 (3.7–33.0)		3	2	3	1–5	
*Ascaris suum*	female	3/33	9.1 (2.4–24.3)	χ^2^ = 0.5 *p* = 0.50	2	2	3	1–3	-
	male	1/23	4.4 (0.01–22.7)		2	2	2	2–2	
Nematodes total	female	5/33	15.2 (6.2–31.4)	χ^2^ = 0.05 *p* = 0.82	3	3	3	1–4	U = 7.0 *p* = 1.00
	male	3/23	13.0 (3.7–33.0)		4	3	3	1–7	

n—number of infected animals; N—total number of animals included in the study. * OPG—oocyst per gram (protozoa) or number of adult parasites in the host (nematodes). GM—geometric mean.

**Table 4:** The prevalence and intensity of gastrointestinal parasite infection in wild boars in relation to body weight.

Parasite	Body Weight (kg)	n/N	Prevalence (%) (95% CI)	χ^2^ Test Value	Intensity of Infection *				
					Mean	GM	Median	Range	Mann–Whitney U-Test Value
Eimeria									
*E. debliecki*	< 40	6/37	16.2 (7.3–31.5)	χ^2^ = 0.2 *p* = 0.65	533	490	400	300–900	U = 0.0 *p* = 0.009
	40–70	4/19	21.1 (8.0–43.9)		125	119	100	100–200	
*E. suis*	< 40	5/37	13.5 (5.4–28.5)	χ^2^ = 1.4 *p* = 0.24	680	673	600	600–800	U = 0.0 *p* = 0.007
	40–70	5/19	26.3 (11.5–49.1)		170	162	150	100–250	
*E. polita*	< 40	3/37	8.1 (2.1–22.0)	χ^2^ = 0.09 *p* = 0.76	350	269	300	100–650	U = 0.0 *p* = 0.20
	40–70	2/19	10.5 (1.7–32.6)		50	50	50	50–50	
*E. scabra*	< 40	3/37	8.1 (2.1–22.0)	χ^2^ = 0.8 *p* = 0.38	583	574	600	450–700	U = 0.0 *p* = 0.10
	40–70	3/19	15.8 (4.7–38.4)		67	63	50	50–100	
*Isospora suis*	< 40	3/37	8.1 (2.1–22.0)	χ^2^ = 1.6 *p* = 0.20	217	208	200	150–300	-
	40–70	0/19	0.0 (0.0–19.8)						
*Eimeria* total	< 40	11/37	29.7 (17.4–45.9)	χ^2^ = 4.2 *p* = 0.04	914	860	800	550–1800	Z = 3.94 *p* < 0.001
	40–70	11/19	57.9 (36.2–76.9)		150	135	150	50–250	
Nematodes									
*Oesophagostomum dentatum*	< 40	3/37	8.1 (2.1–22.0)	χ^2^ = 0.09 *p* = 0.76	2	2	3	1–3	U = 0.0 *p* = 0.20
	40–70	2/19	10.5 (1.7–32.6)		5	4	5	4–5	
*Ascaris suum*	< 40	0/37	0.0 (0.0–11.2)	χ^2^ = 8.4 *p* = 0.004					-
	40–70	4/19	21.1 (8.0–43.9)		2	2	3	1–3	
Nematodes total	< 40	3/37	8.1 (2.1–22.0)	χ^2^ = 3.4 *p* = 0.06	2	2	3	1–3	U = 4.5 *p* = 0.39
	40–70	5/19	26.3 (11.5–49.1)		4	3	3	1–7	

n—number of infected animals; N—total number of animals included in the study. * OPG—oocyst per gram (protozoa) or number of adult parasites in the host (nematodes). GM—geometric mean.

## Data Availability

The data presented in this study are available on request from the corresponding author.
